# Applying the COM-B behaviour change model to a pilot study delivering volatile pyrethroid spatial repellents and insecticide-treated clothing to forest-exposed populations in Mondulkiri Province, Cambodia

**DOI:** 10.1186/s12936-023-04685-1

**Published:** 2023-09-01

**Authors:** Ingrid Chen, Dyna Doum, Kylie Mannion, John Hustedt, Siv Sovannaroth, David McIver, Michael Macdonald, Neil Lobo, Allison Tatarsky, Margaret A. Handley, Josselyn Neukom

**Affiliations:** 1 Malaria Elimination Initiative, Institute for Global Health Sciences, University of California, San Francisco, USA; 2 Health Forefront Organization, Phnom Penh, Cambodia; 3 Menzies School of Health Research, Charles Darwin University, Casuarina, Australia; 4 National Center for Parasitology, Entomology and Malaria Control, Phnom Penh, Cambodia; 5 Innovative Vector Control Consortium, Liverpool, UK; 6 University of Notre Dame, Notre Dame, IN USA

**Keywords:** Forest malaria, Cambodia, Malaria elimination, High risk population, Insecticide treated clothing, Spatial repellent, Volatile pyrethroid, Passive emanator, COM-B model, Behaviour change theory

## Abstract

**Background:**

Southeast Asia is making tremendous progress towards their 2030 malaria elimination goal but needs new interventions to stop forest malaria. This study trials two new vector control tools, a volatile pyrethroid spatial repellent (VPSR) and insecticide-treated clothing (ITC), amongst forest-exposed populations in Mondulkiri Province Cambodia to inform their potential use for eliminating forest malaria.

**Methods:**

21 forest-exposed individuals were given a questionnaire on their perceptions of malaria and preventive practices used, after which they trialed two products sequentially. Clothes was treated with ITC by the study team. Mixed methods were used to understand their experience, attitudes, and preferences regarding the products trialed. Quantitative data was summarized and qualitative insights were analysed using thematic analysis, applying the Capability, Opportunity, and Motivation Behaviour Change (COM-B) model and Behaviour Change Wheel Framework to identify intervention functions to support tailored product rollout amongst these populations.

**Results:**

Study participants reported a need for protection from mosquito bites in outdoor and forest-exposed settings and perceived both products trialed to be effective for this purpose. The VPSR product was preferred when travel was not required, whereas ITC was preferred for ease of use when going to the forest, especially in rainy conditions. COM-B analysis identified that key enablers for use of both products included their perceived efficacy and ease of use, which required no skill or preparation. For barriers to use, the odour of ITC was sometimes perceived as being toxic, as well as its inability to protect uncovered skin from mosquito bites, while the perceived usefulness of the VPSR product trialed was limited by its water sensitivity in rainy forest settings. Intervention components to encourage appropriate and sustained use of these products include education about how to use these products and what to expect, persuasion to use them from community leaders and targeted channels, and enablement to facilitate convenient and affordable access.

**Conclusion:**

The rollout of VPSRs and ITC amongst forest-exposed populations can be useful for eliminating malaria in Southeast Asia. Study findings can be applied to increase product uptake among forest exposed populations in Cambodia, while manufacturers can aim to develop products that are rainproof, easy to use in forest settings, and have favourable odour profiles to target users.

**Supplementary Information:**

The online version contains supplementary material available at 10.1186/s12936-023-04685-1.

## Background

Southeast Asia strives to eliminate malaria by 2030 and has made tremendous progress even during the COVID-19 pandemic [1]. Although reported malaria cases have declined by 81% between 2000 and 2021 [1, 2], the remaining pockets of transmission will be the most difficult to address, mostly located in remote forested locations where local *Anopheles* vectors are highly diverse, with primary vector species including outdoor and day-biting vectors, such as *Anopheles dirus, Anopheles minimus,* and *Anopheles maculatus* [3–7]. The biting habits of these mosquitoes present challenges to elimination efforts, resulting in residual transmission, which refers to vector biting habits that occur despite the use of traditional vector control interventions, such as insecticide-treated nets and indoor residual spraying.

Research and development efforts to address residual malaria transmission in forest settings has been slow, with long-lasting insecticidal hammock nets (LLIHN) being rolled out on an emergency basis led by the Regional Artemisinin-resistance Initiative (RAI), with available evidence suggesting that hammock nets offer some protection against vector biting behaviour [8–10]. However, even when hammock nets are used, there is a need for additional interventions to address residual transmission when people at risk are not sleeping or resting, both indoors and outdoors. Potentially promising vector control products for forest-exposed individuals when awake, whether working or socializing, include insecticide-treated clothing (ITC), volatile pyrethroid spatial repellents (VPSRs), and/or topical repellents, which may be supplemented with chemoprophylaxis and/or endectocide use to offer additional protection [11–13].

This study is part of a multi-stage assessment of new products for forest-exposed populations in Cambodia and the Greater Mekong Subregion that intends to support Cambodia’s 2025 malaria elimination goals [14, 15]. In this pilot study, forest goers who gather wood, mushrooms, and/or other products in the forest, dwellers who live in or near the forest, and rangers supported by the government and various non-governmental organisations were characterized and given VPSRs and ITC. Their perceptions of malaria, gaps in protection from mosquito bites, experience trialing both products piloted compared to current practices in use, recommended messaging around each product, and communication channels where they seek information for malaria prevention and care are described. Findings are then applied to the Capability, Opportunity, and Motivation Behaviour Change (COM-B) framework and the related Behaviour Change Wheel (BCW) [16] to inform the design of behaviour change interventions for rollout of these products among these populations in an implementation research study. Pending validation in larger studies, findings from this study can also be applied to guide the user-centred design of future products that support the elimination of forest malaria.

## Methods

### Overview and study design

**Fig. 1:**
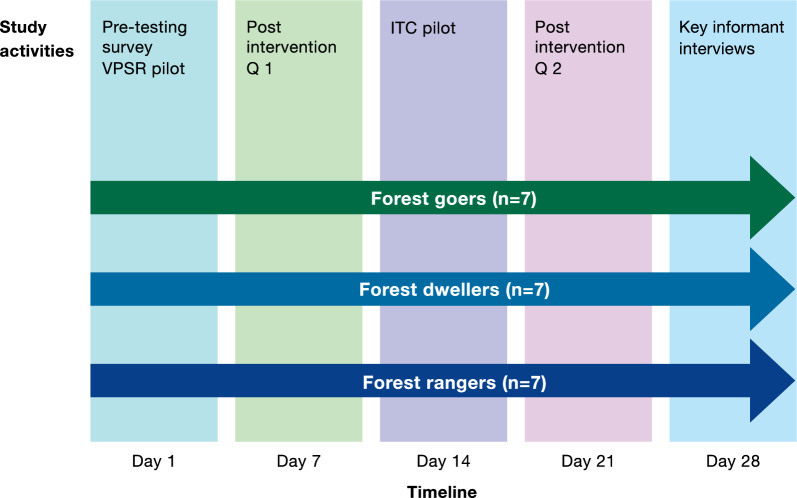
Study timeline

A prospective cross-over study was conducted among a convenience sample of forest exposed individuals, undertaking a mixed methods approach in order to identify factors that can enhance uptake of the mosquito bite prevention products using a behaviour change theory-informed approach (see below). Participants were interviewed prior to trialing products to establish demographics, perceptions of malaria, working and social habits, and mosquito bite prevention practices and preferences. After this pre-trial questionnaire was conducted, participants were given the VPSR product to trial for 7 days (Fig. 1). A quantitative survey was then conducted to assess the perceived functionality, tolerability, usability, and user acceptability of the VPSR product, followed by a 7-day trial of the ITC product where clothes were treated by the study team and user experience was then assessed using the same quantitative survey. Key informant interviews were then conducted to understand the overall experience and attitudes towards and between the two products trialed.

### Study location and population

**Fig. 2:**
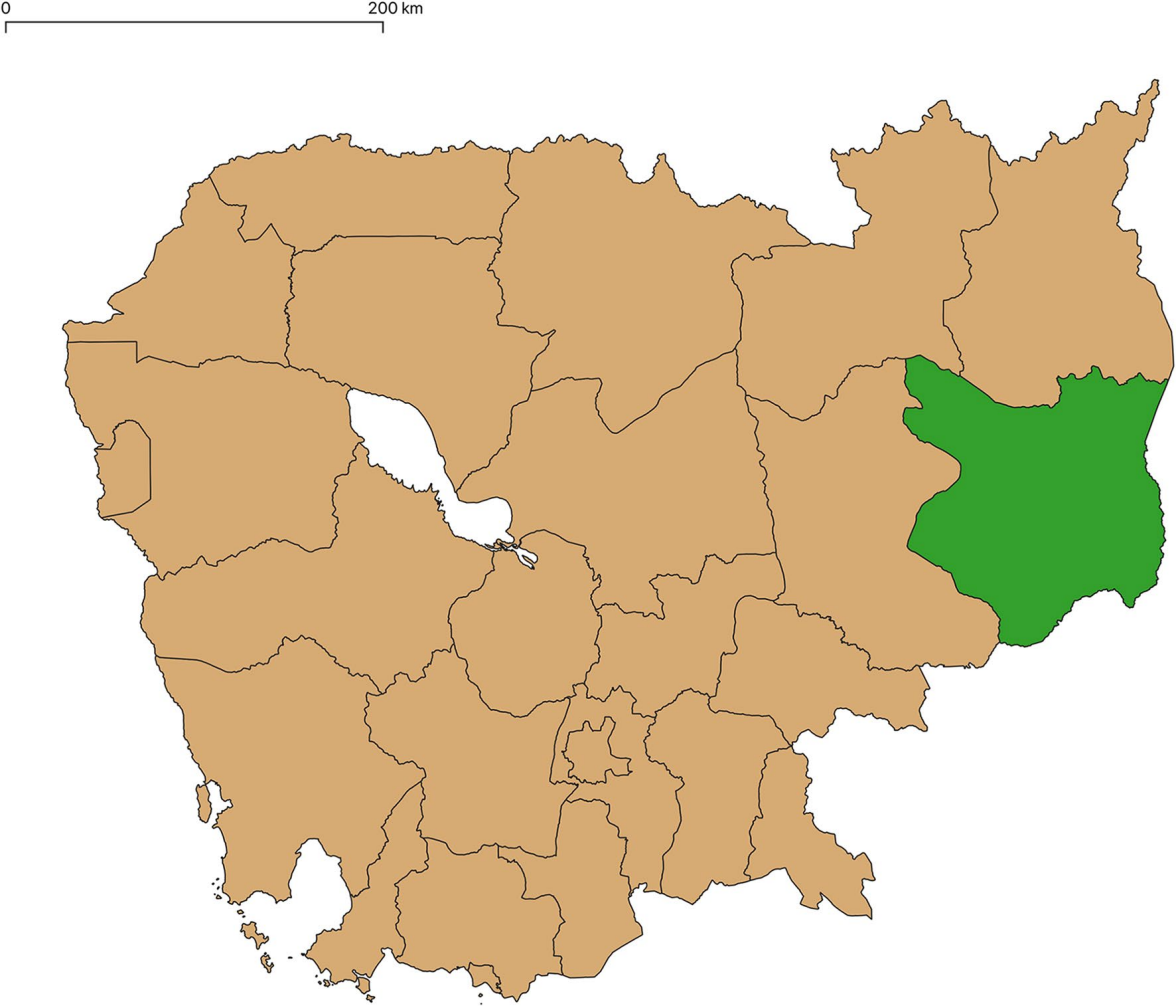
Map of Cambodia, Mondulkiri Province in green

This study took place in Mondulkiri Province, Cambodia (Fig. 2), where malaria transmission occurs year-round, with higher case rates occurring during the rainy season from August to January. A mixture of villages from different areas of Mondulkiri Province were considered, to ensure maximum variability in environmental factors and mosquito bite prevention practices and preferences in the sample. Villages and locations selected for recruitment were selected based on proximity to forested areas, high malaria incidence according to 2021 government data, and recommendation by the Provincial Health Department. The study population (n = 21) included three high-risk populations for malaria: forest rangers (n = 7), forest goers (n = 7), forest dwellers (n = 7), and forest rangers (n = 7), the latter two of which were classifications of risk groups created based on consultation with locals [6, 7, 17–20]. These risk groups were created in order to examine whether they were different enough to be classified separately in future forest malaria studies, both in this pilot study and in the larger parent study. Forest goers were defined as those living in forest fringes who travelled to the forest regularly for seasonal farming, hunting, or foraging for mushrooms, vegetables, and resin, as well as seasonal forest workers who migrate for gem mining, logging, and work in plantations, usually men. Forest dwellers were defined as those living in the forest or less than 1 km from the edge of the forest permanently, surviving on subsistence farming within the forest. Many forest dwellers have two living structures; a house in the village that they live in during part of the year, and a more open temporary structure in the farm or forest where they live during planting and harvesting seasons.

Forest rangers are individuals working for government or wildlife and conservation agencies to protect the forest and areas near international borders, the vast majority of whom are men. Rangers are required to spend 16 nights per month in the forest on patrol. Although they have ranger stations available for sleeping, which can be along main roads or further into the forest, they also sleep in hammocks near rivers or villages where they are working. In addition to being provided with salary, ranger employers are also provided with food and mosquito bite prevention tools including government issued hammock nets, mosquito coils, and optional topical repellents, the latter of which was provided every 1–2 months. Also, sometimes rangers received donated products such as topical repellent lotions and insecticide sprays from their employers, the Ministry of Health, and implementing partners supporting elimination efforts.

### Inclusion criteria

Participants eligible for inclusion were those who reported living in or visiting forested areas (at least 1 day per week on average), who expressed an intention to stay in these locations for the next month while products were being piloted. All participants were ≥ 18 years of age and reported being fluent speakers of Khmer and/or Bunong.

### Recruitment

A member of the study team (DD) working for the Health Forefront Organization conducted recruitment for the study. To recruit forest goers and dwellers from these villages, the study team and local health centre staff members met with the village chief to inform them of the study, its procedures, and its objectives. The village chief identified individuals who would likely be eligible for inclusion for the study, liaised with those individuals, and scheduled meetings between individuals interested to partake in the study and the researcher. During this scheduled meeting, the study team introduced themselves to prospective participants and checked if they met the study inclusion criteria. For those eligible and interested to partake in the study, detailed information about the study was provided, informed consent was sought, and a one-hour meeting for the pre-trial questionnaire was scheduled either immediately or later that day.

To recruit forest rangers who lived in or near the villages selected for recruitment, the study team contacted the director of wildlife sanctuary, requesting permission to enter restricted areas to meet forest rangers for research purposes. Permission was granted, after which a time was arranged for the study team to meet with the rangers at a ranger station. At this meeting, information on the study was provided, informed consent was sought, and a one-hour meeting for the pre-trial questionnaire was scheduled either immediately or later that day.

### Products piloted

Two new vector control products were piloted in this study: a transfluthrin-based VPSR, and an etofenprox-based insecticide formulation (Perimeter ETO Insect Guard formulation) used for treating clothing, described further below. All individuals enrolled in the study were instructed on the appropriate and safe use of products by the study team, including a live demonstration, before being asked to use each product for 7 days indoors and outdoors, at any time. The VPSR was piloted by all participants first, after which ITC was then piloted. Participants were instructed that during the trial period that they should use these products along with any vector control tools they were already using.

The passive VPSR included the odourless active ingredient transfluthrin (manufactured by Bayer) which has an excellent safety profile in mammals [21] and can prevent mosquito biting and induce mosquito mortality [22, 23], depending on their levels of exposure to this active ingredient. For the product piloted, transfluthrin was infused in 2 small, rectangular lightweight substrates approximately the size of an A4 piece of paper (size 8.25 × 11.75 inches) and hung up to create an area of protection from insect bites. In this study, this VPSR product was given to users who were told that they should handle the product when wearing latex gloves that were provided with the product. This specific VPSR product was designed for indoor use, but for the purposes of this research study, participants were told that they could use the product indoors or outdoors as long as they avoided getting the product wet.

The insecticide used to treat clothing in this study was etofenprox, manufactured by Mitsui Chemicals, in a proprietary formulation developed by Pine Belt Processing, a wholly owned subsidiary of Warmkraft, Inc. Etofenprox is a United States (US) Environmental Protection Agency (EPA)-approved insecticide developed to treat clothing worn by the US military. In this study, the researcher and assistant used the product to treat both forest ranger uniforms and the every-day civilian clothing provided by forest goer and dweller participants, providing a demonstration of how to treat the products. Participants observed this process but did not treat their clothes themselves.

### Data collection and analysis

This study had four components:

1. Pre-trial questionnaire: (Additional file 1) Established the demographics, perceptions of malaria, working and social habits, and mosquito bite prevention practices and preferences of study participants. The two researchers who sought informed consent were present at each interview; one researcher conducted the interview using the questionnaire in Open Data Kit (ODK) with smart phones to limit data entry errors, and the other took notes. This process was not audio or video recorded. After each interview was completed, data were upload into a cloud server. Once data collection was complete, data were downloaded onto a password secured laptop, and consolidated in Microsoft Excel (Microsoft Corporation Version 2202). A Khmer version and an English version of survey responses were provided, and the English database was sent to a researcher (IC), who analysed the data using Stata (StataCorp Version 17.0).
2. Post-trial questionnaire: After using each product for 7 days, all individuals enrolled in the study completed a post-trial questionnaire (Additional file 2) on their experiences, preferences, and attitudes toward the use and functionality of that product including tolerability, usability, and user acceptability. A Likert scale was used, which presents several options on how much participants agree or disagree with specific statements, to assess their understanding on the use of the products, their overall usefulness and frequency of use, perceived changes to the number of mosquito bites after use, side effects or discomfort, preference of these products over other products in use, willingness to pay for products monthly, and locations preferred for purchase. The interviews were conducted in Khmer by two members of the study team, one of whom administered the interview and the second of which took notes. Once data collection was complete, analysis was conducted as described for the pre-trial questionnaire.
3. Key informant interviews: After piloting both products, an interview guide (Additional file 3) was used to ask participants about their overall experience and attitudes toward and between the two vector control products trialed, their preferences relative to other mosquito bite prevention tools used prior to the study, and about factors that might influence use. They were also asked about recommended messaging about each product, and which communication channels they recommended using for disseminating information on malaria prevention and care.
  The same two researchers who conducted the questionnaire conducted the interviews, one of whom took notes. The interviews were audio-recorded, and responses to each question were collated in Microsoft Word (Microsoft Corporation Version 2302).
  The two interviewers transcribing interviews found results between all 21 interviews to be very similar, and after translating a random selection of ten interviews into English, agreed that because results among all participants interviewed were so similar, these 10 interviews were sufficient for informing the COM-B analysis of the pilot study. One researcher (IC) reviewed translated results from the ten interviews and agreed that results were very similar to one another, and proceeded to conduct thematic analysis using those ten interviews comprising results from 2 forest dwellers, 5 forest goers, and 3 forest rangers. The same researcher (IC) transposed the results into Microsoft Excel (Microsoft Corporation Version 2202), reviewed the results, and agreed with the two interviewers that responses amongst participants were similar, and that theoretical saturation had been reached. Themes on the findings from each question or set of similar questions were identified by that researcher, who reviewed the translated results three times to confirm findings, then highlighted representative quotes from each theme. The interviewer and assistant reviewed results and discrepancies were resolved through review of the original data.
4. *COM-B analysis****.*** Results from (2) and (3) were analysed by three researchers (IC, MH, and JN) using the COM-B framework to identify barriers and enablers to product uptake, in this case the behaviours were to use the vector control products in addition to any vector control tools they were already using (e.g., long-lasting insecticidal net (LLIN), LLIHN) [15]. The COM-B behaviour change model has been used across a wide range of topics to collect community input on health behaviour intervention development [15, 24, 25]. Capability refers to the perceived ability to engage in the physical processes and thoughts necessary to use the intervention, opportunity refers to social and environmental influencers in the settings being studied, and motivation refers to individual beliefs, emotions, and impulses that influence behaviour, but may not be consciously recognized [15]. After barriers and enablers to wearing/using the products were identified using COM-B, they were mapped to specific intervention components to consider, using the BCW [14]. The BCW is an extension of the COM-B model that allows for each potential barrier and enabler to be linked to an intervention function that can allow for a change in behaviour to be achieved (e.g., enablement, modelling, education). It also incorporates methods for the selection of contextually appropriate intervention components and options to consider for delivery of the selected approaches [14, 15]. Based on COM-B results, priority intervention components were selected to allow for the trial distribution of these products, as well as sustained delivery, due to their relevance for informing implementation approaches.

#### Sample size calculation

The sample size for this study was based on the quantity of vector control products available. The products piloted were still in development and not yet available at large scale that normally follows commercialization. The quantities available allowed for 21 individuals to pilot these products at this stage of the research programme.

## Results

### Demographics

Participants of this study were mostly men, aged 26–35, consisting of a mixture between Khmer and Bunong ethnic groups (Table 1). All participants lived in or near the forest, with forest dwellers and goers mostly being farmers who cultivate rice, cassava, cashews, and other crops as their main source of earnings. For ethnicity, all forest rangers were of the Khmer ethnic group, whereas forest dwellers and forest goers comprised both Khmer and Bunong ethnic groups. All participants spoke the Khmer language fluently and most could read or write it. Most could also speak Bunong fluently, although only three forest goers could read or write in this language, the written form of which has only existed in recent decades. For levels of education, the forest rangers enrolled generally had higher levels of education, while forest dwellers had less.

**Table 1:** Participant demographics (n = 21 for post-intervention questionnaire, n = 10 for key informant interviews)

Characteristic	Post-intervention questionnaire				Key informant interviews			
	Total (%)	Forest goers	Forest dwellers	Forest rangers	Total (%)	Forest goers	Forest dwellers	Forest rangers
Total individuals (n)	21 (100%)	7	7	7	10 (100%)	5	2	3
Age (years)								
18–25	3 (14%)	2	0	1	2 (10%)	1	0	1
26–35	9 (43%)	3	3	3	2 (20%)	2	0	0
36–45	3 (14%)	1	1	1	3 (30%)	1	1	1
> 45	6 (29%)	1	3	2	3 (30%)	1	1	1
Gender								
Male	15 (71%)	7	1	7	8 (80%)	5	0	3
Female	6 (29%)	0	6	0	2 (20%)	0	2	0
Ethnic group								
Khmer	11 (52%)	1	3	7	4 (40%)	1	0	3
Bunong	10 (48%)	6	4	0	6 (60%)	4	2	0
Languages								
Khmer								
Understand spoken	21 (100%)	7	7	7	10 (100%)	5	2	3
Speaking (fluent)	21 (100%)	7	7	7	10 (100%)	5	2	3
Reading	18 (86%)	6	5	7	7 (80%)	0	4	3
Writing	18 (86%)	6	5	7	7 (70%)	0	4	3
Bunong								
Understand spoken	17 (81%)	7	5	5	9 (90%)	2	5	2
Speaking (fluent)	15 (71%)	5	5	5	7 (70%)	2	3	2
Reading	3 (14%)	3	0	0	2 (20%)	0	2	0
Writing	3 (14%)	3	0	0	2 (20%)	0	2	0
Highest level of education								
Completed high school	2 (10%)	0	0	2	1 (10%)	0	0	1
More than secondary	2 (10%)	2	0	0	1 (10%)	1	0	0
Completed secondary	1 (5%)	0	0	1	0 (0%)	0	0	0
Some secondary	5 (24%)	1	2	2	2 (20%)	1	0	1
Some primary	8 (38%)	4	2	2	4 (40%)	3	0	1
None	3 (14%)	0	3	0	3 (30%)	0	2	0

### Pre-trial questionnaire

#### Perceptions of malaria and care-seeking behaviour

When asked about their perceptions of malaria, individuals were presented with options on whether they agreed or disagreed with a series of questions. Responses were similar between all participant groups; all agreed that mosquito bites are dangerous, that they worried about mosquito bites, and that mosquito bites cause itching and make ugly marks on the skin. Most participants agreed that mosquito bites can cause severe disease needing hospitalization. The reasons for worrying about mosquito bites included getting sick with malaria and dengue, as cited by forest rangers and goers, as well as the costs of going to the hospital, as cited by forest dwellers, the group that described the most substantial barriers to accessing care.

When asked about malaria diagnosis and care-seeking behaviour, the most common method cited for diagnosis was using microscopy or a malaria rapid diagnostic test. Care was sought in private clinics (76%), referral government hospitals (71%), government health centres (48%), or from the village malaria worker (VMW, 5%). Forest rangers typically visited government hospitals, while goers and dwellers did not have a main source of care, reporting that they went to a variety of these facilities. When asked about what they did to recover from malaria, all mentioned taking medicine, with 6 participants mentioning that in addition to medicine, they received intravenous therapy.

#### Working and social habits

All participants stated that they spent time in the forest, with forest rangers spending 4–5 days a week in the forest, presumably for work, while forest dwellers and goers reported working or travelling in the forest 3–4 days a week. All participants reported going to the forest with others; forest rangers and dwellers went with co-workers, while forest goers reported going with neighbors. Participants seldom reported going deep into the forest with their family members.

**Fig. 3:**
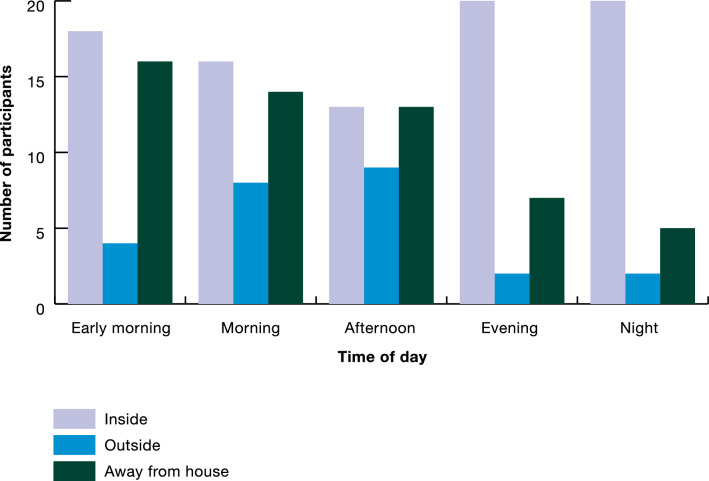
Locations where participants spend time

When asked about where they spent their time during the day, all individuals reported waking up before 7 AM and going to sleep around 9 PM, spending time inside the house, outside of the house, and away from the house throughout the day (Fig. 3). Responses were similar among all participant groups, with rangers reporting more time spent away from their house (the ranger station) during all of the times that they were asked about.

#### Mosquito biting frequency, times, and locations

**Fig. 4:**
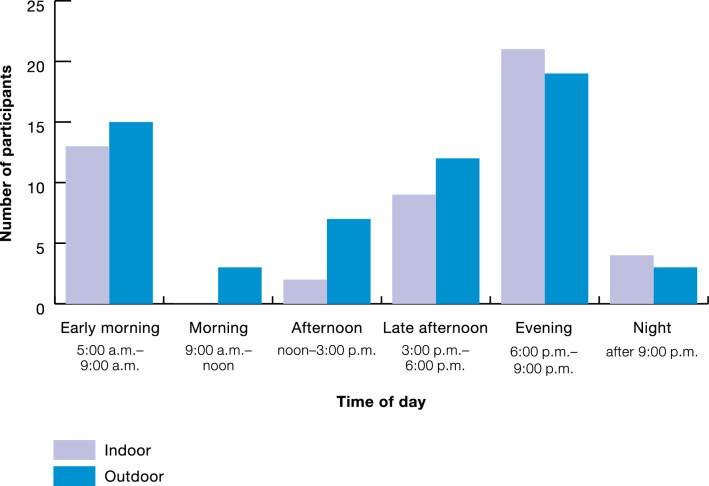
Perceived mosquito biting times

When asked about being bitten by mosquitoes inside or outside of their houses, all participants stated they had been bitten by a mosquito recently, with approximately half reporting being bitten inside the house the day they were interviewed, and the other half saying they were bitten the day before. These self-reported biting frequencies were similar inside the house and outside the house, with biting times shown in Fig. 4.

Perceived mosquito biting times were similar amongst all participants in the study, with the most commonly cited biting time being in the evening (6–9 PM), occurring both inside the house and outside of the house. At this time, most forest goers and dwellers reported being inside the house, with rangers being inside, outside, or away from the house (Fig. 3). The second most commonly reported biting time was 5–9 AM, when forest goers and dwellers reported mostly being inside the house and sometimes away from the house, and rangers reporting being inside, outside, or away from the house. The third most commonly reported biting time was late afternoon (3–6 PM), where participants reported being in all locations, with more rangers reporting that they get bitten by mosquitoes during this time.

#### Mosquito bite prevention habits

When asked about which mosquito bite prevention methods participants used, all participants reported using methods to prevent malaria inside their house or ranger station, and in the forest, and most (76%) stated that they used methods to prevent malaria outside the house/ranger station. Some forest goers and dwellers reported not knowing which bite prevention methods to use in forest.

**Fig. 5:**
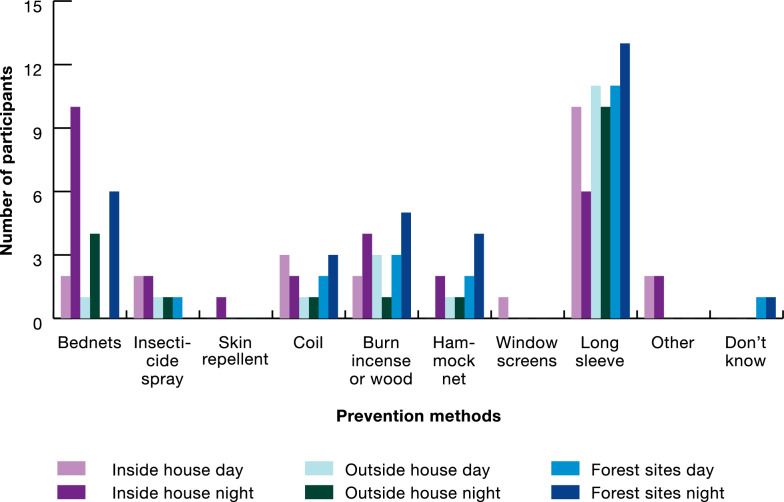
Mosquito bite prevention methods used inside and outside the house and in the forest

A summary of mosquito bite prevention methods used is in Fig. 5 with results shown by participant group in Table 2. The most common methods used indoors were mosquito nets and coils, which were mostly used by forest goers and dwellers. Forest rangers reported using approximately twice the number of mosquito prevention methods as dwellers and goers. These rangers explained they did not use mosquito nets often because they could not be hung in the bunk beds at the ranger station. Instead, rangers described using skin repellents, hammock nets, and insecticide spray which is an aerosol spray that rapidly kills mosquitoes and other insects.

**Table 2:** Bite prevention methods used inside and outside the house and in the forest

Time, location, and intervention used	Total (%)	Forest goers	Forest dwellers	Forest rangers
Inside daytime				
Sleep under mosquito net	7 (33%)	3	4	0
Use insecticide spray	10 (48%)	4	3	4
Use skin repellent	4 (19%)	0	0	4
Burn coil	9 (43%)	5	3	1
Burn incense or wood	1 (5%)	0	1	0
Use hammock net	5 (24%)	1	0	4
Use long sleeve clothing	4 (19%)	1	0	3
I don't know	0%	0	0	0
Inside night time				
Sleep under mosquito net	15 (71%)	6	7	2
Use insecticide spray	7 (33%)	2	1	4
Use skin repellent	4 (19%)	0	0	4
Burn coil	8 (38%)	4	2	2
Burn incense or wood	2 (10%)	1	1	0
Use hammock net	8 (38%)	0	1	7
Use long sleeve clothing	3 (14%)	0	0	3
I don't know	0%	0	0	0
Outside daytime				
Sleep under mosquito net	0%	0	0	0
Use insecticide spray	4 (19%)	1	1	2
Use skin repellent	6 (29%)	0	0	6
Burn coil	1 (5%)	1	0	0
Burn incense or wood	3 (14%)	2	1	0
Use hammock net	0%)	0	0	0
Use long sleeve clothing	11 (52%)	4	4	3
I don't know	0%	0	0	0
Outside night time				
Sleep under mosquito net	2 (10%)	1	1	0
Use insecticide spray	3 (14%)	0	0	3
Use skin repellent	5 (24%)	0	0	5
Burn coil	5 (24%)	2	0	3
Burn incense or wood	3 (14%)	1	0	2
Use hammock net	2 (10%)	0	0	2
Use long sleeve clothing	9 (43%)	3	4	2
I don't know	1 (5%)	0	1	0
Forest daytime				
Sleep under mosquito net	0%	0	0	0
Use insecticide spray	3 (14%)	0	0	3
Use skin repellent	6 (29%)	0	0	6
Burn coil	2 (10%)	2	0	0
Burn incense or wood	5 (24%)	2	2	1
Use hammock net	0%	0	0	0
Use long sleeve clothing	10 (48%)	3	4	3
I don't know	4 (19%)	1	3	0
Forest night time				
Sleep under mosquito net	0%	0	0	0
Use insecticide spray	3 (14%)	0	0	3
Use skin repellent	7 (33%)	0	0	7
Burn coil	7 (33%)	2	0	5
Burn incense or wood	2 (10%)	1	1	0
Use hammock net	7 (33%)	0	0	7
Use long sleeve clothing	12 (57%)	3	4	5
I don't know	6 (29%)	3	3	0

Methods used outside of the house (but near the house) and in forest sites (away from the house) were similar although fewer methods were used in forest sites as compared to other settings. Forest goers and dwellers most commonly reported wearing long-sleeved clothing and burning coils both day and night, outside of the house and in the forest. For forest rangers, methods used during the day were most commonly topical skin repellents, followed by wearing long sleeves and insecticide spray. At night, skin repellents were most commonly used outside the house and in the forest, followed by burning coils, wearing long sleeves, and using insecticide spray. The main notable difference between forest ranger prevention methods used outside of the house vs in the forest at night were hammocks, which were more commonly used in the forest as compared to outside of the house.

When asked about their ownership and usage of bed nets, forest goers and dwellers were similar, all of whom reported that their household owned one to three bed nets. These were obtained from the government free of cost or purchased for 20,000 and 50,000 Riel ($4.86 and $12.15 USD) per net from retail shops or markets 36 to 60 km from their homes (Table 3). Reported bed net usage was high, with all 14 forest dwellers and goers reporting that they used bed nets the last time they stayed in the house, with multiple people sleeping under nets. The questionnaire did not specify whether the bed nets were treated with insecticides or not. The majority of forest goers and dwellers also reported owning at least one hammock net, most of which they believed were treated with insecticides (based on evidence of procurement with support from the National Center for Parasitology, Entomology, and Malaria Control (CNM) or Global Fund). These hammock nets were either provided for free from the government or purchased for 50,000 to 60,000 Reil (USD $12.15 to $14.58) between 15 and 60 km from their homes. Only two hammock nets were reported to be used the night before the interview, inside the house.

**Table 3:** Bed net and hammock use

Characteristic		Total (%)	Forest goers	Forest dwellers	Forest rangers
Total individuals (n)		21 (100%)	7	7	7
Bed nets					
Number owned in household					
0		6 (29%)	0	0	6
1		4 (19%)	2	1	1
2		5 (24%)	3	2	0
3		6 (29%)	2	4	0
Hammock nets					
Number owned in household^a^					
0		2 (10%)	0	2	0
1		14 (67%)	5	2	7
2		4 (19%)	2	2	0
3		1 (5%)	0	1	0

^a^For forest rangers, the household refers to the ranger station

For forest rangers, all owned one hammock, and only one used a bed net obtained from a market 15 km away from the ranger’s home. The hammock nets were obtained free of charge from their work, which are typically a hammock zip net which allows for a rain fly to be hung over the top. It was unclear whether these hammock nets were treated with insecticides. Forest rangers reported the frequent use of hammock nets, with the vast majority stating they used the net the night before they were interviewed, both in the ranger station in the forest and in the forest itself.

### Post-trial questionnaire

After the pre-intervention questionnaire, participants piloted the VPSR for 7 days and were then administered a questionnaire. After that questionnaire, participants tried the ITC for 7 days and were given the same questionnaire. Results are summarized below in Table 4 and described for VPSR, followed by ITC.

**Table 4:** User experience with VPSR and ITC (n = 21)

Participant experiences with product	Intervention and number (%)	
	VPSR	ITC
Understood specific use of product		
Yes	21 (100%)	20 (95%)
No	0	1 (5%)
Overall rating of product		
Very useful	15 (71%)	2 (10%)
Useful	6 (29%)	12 (57%)
Not very useful	0	3 (14%)
Don’t know	0	4 (19%)
How often product was used		
At least once a day	21 (100%)	16 (76%)
Every 2 days	0	1 (5%)
Never used	0	4 (19%)
Perceived changes to number of mosquito bites after use		
Many fewer bites	19 (90%)	3 (14%)
Moderately fewer bites	2 (10%)	12 (57%)
No change noticed	0	2 (10%)
Don’t know	0	4 (19%)
Side effects or discomfort		
Yes		
Pain on skin	1 (5%)	0
Foul smell	0	17 (81%)
Itchiness	0	7 (33%)
Skin irritation	0	2 (10%)
Dizziness	0	2 (10%)
Headache	0	4 (19%)
No	20 (95%)	4 (19%)
Preference of this product over other products		
LLINs	12 (57%)	4 (19%)
Hammock nets	11 (52%)	2 (10%)
Skin repellents or mosquito coils	15 (71%)	9 (43%)
Willingness to use product if there is access		
Yes	21 (100%)	13 (62%)
No	0	5 (24%)
Don’t know	0	3 (14%)
Willingness to pay for product^a^ (USD per month)		
$0.49–1.23	8 (38%)	3 (14%)
$1.24–2.95	11 (52%)	5 (24%)
$2.96–4.92	2 (10%)	3 (14%)
Locations preferred for purchase		
Market	15 (71%)	8 (38%)
Health center	12 (57%)	8 (38%)
Mobile malaria worker	0	2 (10%)
Shop in village	13 (62%)	4 (19%)
Nearest market	8 (38%)	8 (38%)
Mobile or moto seller	2 (10%)	0
Private doctor or clinic	1 (5%)	0
Other	1 (5%)	0

^a^Based on conversion of 4,066 Reil/USD (exchange rate June 23, 2022)

#### VPSR

All participants used the VPSR product during the piloting phase and found it useful or very useful and perceived reductions in mosquito bites after using it. They reported using the VPSR indoors, in the bedroom or in the main living area, and some participants, mostly forest rangers, brought the VPSR to the forest with them in their backpack. Those who did not carry the VPSR product to the forest stated that they were concerned about the product getting wet from the rain. Generally, participants liked the odourless quality of the VPSR as well as how the product looked. Only one side effect was reported by a forest ranger who touched the product without gloves, mentioning that he felt pain on his skin similar to a needle injection.

When asked about whether they preferred the VPSR to other mosquito bite prevention methods they were using, most preferred the VPSR to LLINs, hammock nets, and skin repellents or mosquito coils, due to its perceived ability to chase mosquitoes away, both indoors and in the forest. Other preferable aspects of the VPSR included that it was more comfortable to sleep with compared to LLINs which can be hot, and did not create the bad smell and smoke generated when using mosquito coils which participants perceived to be harmful to their health. On the other hand, some participants mentioned preferring LLINs to the VPSR product due to their long duration of efficacy and the physical barrier they provided from mosquitoes. They also preferred hammocks and skin repellents due to their ability to withstand rain, which they mentioned was particularly helpful when spending time in the forest. All participants stated that they would recommend the VPSR product to others and would be willing to pay for it, with willingness to pay most commonly amounting to 5000 or 10,000 Reil ($1.22 or 2.44 USD) monthly.

#### ITC

Most participants (81%) used the ITC; those who did not pilot the product were concerned about its smell, which they believed meant that the product was unsafe to use. Those who used ITC reported wearing it to the forest and some found the product to be useful, perceiving moderate reductions in mosquito bites during its use. However, several side effects were noted while wearing ITC. Most participants mentioned its unpleasant odour, and some also noted that wearing the treated clothes caused itchiness, skin irritation, and/or dizziness.

When asked about whether they preferred the ITC as compared to LLINs, hammocks, coils, and skin repellent, responses varied widely. Forest rangers preferred treated clothes to LLINs and hammocks, mentioning their usefulness when spending time in the forest. Those who preferred LLINs and hammocks believed that these tools offered better protection than ITC, had longer durations of efficacy, were more useful for sleeping, and did not have the bad smell that the treated clothes had. Forest rangers generally agreed that they would recommend this product to others, while other participant groups gave mixed responses. Those who would recommend the product expressed that they would be willing to pay on average 11,000 Reil ($2.71 USD) monthly, while those unwilling to pay stated their reasons as a perceived lack of efficacy, bad smell, and that the product caused itchy skin.

### Key informant interviews and COM-B analysis

Data from a convenience sample of ten key informants comprising two forest dwellers, five forest goers, and three forest rangers is presented below, with their demographics shown in Table 1. Themes on perceptions of malaria (Table 5) and experience trialing VPSR and ITC were summarized (Table 6), including application of the COM-B framework to code for barriers and enablers to product use.

**Table 5:** Health beliefs related to malaria prevention and care

Theme	Quotes	COM-B code
Malaria is risky to myself and my community members because it is a disease that can be deadly and because there are limited measures of protection available for use when I am in the forest. Forest exposed individuals cannot work if they are sick with malaria, and for forest rangers this limits their ability to achieve their mission to protect the forest	“*[We have a] big risk because almost everyone in the community works in the forest or stays at the house in the forest, the back of the house is covered by forest. [We have] complete exposure to the risk of getting malaria*.”—Female forest dweller, age 55 *“I’m worried about my forest ranger colleague and the people in the community as well, because we live in the forest and we go to work together. If anyone is sick then our work is stuck.”*—Male forest ranger, age 24	Enabler: psychological motivation Perceived threat of malaria can motivate the desire to try out new products Enabler: reflective motivation and social opportunity Desire to protect one’s family and colleagues can encourage the use of these products. For rangers specifically, their dedication to their work mission can also motivate product —use
Spending time in the forest poses risks to malaria infection, particularly of concern for children and forest goers who are perceived as most vulnerable populations	“*Forest goers and potato collectors are getting malaria and suffering from it most.*”—Male forest goer, age 46 *“Children [get malaria often] because they go look after cows in the forest and do not protect themselves well from mosquito bites.*”—Male forest goer, age 31	Enabler: automatic motivation Concern for vulnerable groups may increase the uptake of preventive products
It is difficult to access care for malaria due to the costs involved and distance from sources of test and treatment	“*I work in the forest most of this time, this disease can cause death if not treated. Every time I get sick I need to spend money, which is difficult to find*”—Male forest goer, age 26 “*During the rainy season it is difficult to see health staff and village malaria workers for a blood test because we live in the forest far from villages and health centers*”—Male forest ranger, age 51	Enabler: reflective motivation Perception that products can prevent seeking healthcare can motivate their use
We need methods to protect ourselves from mosquito bites when spending non—resting time in the forest	“*I go to the forest and fields in the forest where there are lot of mosquitoes, I do not have proper protection, only from sleeping in the hammock*.”—Male forest goer, age 26	Enabler: psychologic motivation and automatic motivation Products piloted are seen as meeting a need that is not met by the other prevention products available because they offer protection from malaria and dengue fever during non-resting time in the forest

**Table 6:** Key informant perceptions common to both products piloted

Theme	Quotes	COM-B code
We want both products piloted to be available to our communities because it offers us protection from malaria	*“[People] generally like all methods that can protect against mosquitoes… I want others in the village to use [these products] as well, because this product could stop mosquito bites. I want them to be safe and healthy.”*—Male forest goer, age 31 “*[I want to tell my community about] the effectiveness of [these products]. If it is effective, easy to use, and has good quality, I don't need to explain much to them, they will see the effect and everyone will want to have it. People are afraid to get sick*.”—Male forest goer, age 26 “*[The treated clothes] reduces mosquitoes and prevents malaria and dengue… I recommend [that my colleagues use] the product when they go to the forest too, according to my experience using it*.”—Male forest ranger, age 51	Enabler: psychological capability and reflective motivation Perception that using the product will be easy and at the same time, provide protection from malaria for one’s family and community
Both products were efficacious for keeping mosquitoes away	*“[I thought the VPSR product was helpful because] when I hung the product inside my house, I didn't see mosquitoes inside my house and no mosquitoes bite both day and nighttime*”—Female forest dweller, age 55 “*I recommend the team to use [ITC] because the mosquitoes do not dare to come near us.*”—Male forest ranger, age 37	Enabler: psychological capability Users see that the product is efficacious
Users like that both products were easy to use	“*[The VPSR product was] easy to use, fast to install, efficacious.”*—Male forest ranger, age 51 *“[The VPSR product is] nice and lightweight”*—Female forest dweller, age 55 “*[The ITC are] easy to use, just wear clothes that are safe from mosquito bites*.”—Male forest goer, age 23	Enabler: physical capability and automatic motivation Products were easy to use and users liked them
The smell of the products affects their efficacy and safety	“*I didn’t notice any smell [for the VPSR], I can't believe that just a kind of paper without any smell can take the mosquitoes away. It is real that the product works well.*”—Male forest goer, age 26 “*[The treated clothes] prevents mosquito bites, there was some smell and the smell made the mosquitoes uncomfortable and ran away from us*.”—Female forest dweller, age 40 *“[I was] worried about poisoning and getting sick and going to the hospital even after the project staff explained to me. I am concerned because of the way the clothes smell after treatment, that's why I wash them to reduce the smell.*”—Male forest goer, age 31	Barrier: psychological capability and automatic motivation Misperception about odor, such that there was discomfort and concern that smelly products might be toxic and that odorless ones might not be effective
People do not like spending money on preventive products for malaria	“ *[People] don't like spending money, for example buying mosquito coils*.”—Male forest goer, age 26	Barrier: physical opportunity Resources to buy the products may not be available

#### Health beliefs and perceptions of malaria

Consistent with results found from the pre-trial questionnaire, participants expressed that they were worried about malaria, recognizing the risks it posed to themselves and their community due to the amount of time they spend in the forest, which is an enabler to product use (Table 5). Additional enablers to product use included their ability to protect vulnerable populations, such as children, and that preventing malaria infection could avert treatment-seeking costs which are a particular challenge owing to the remote locations in which these participants live.

#### Experience with piloting VPSR and ITC

Participants identified positive attributes associated with both products trialed. Common themes applying to both products trialed are described in Table 6, while attributes where the products differed, as well as participant comparisons between the products are described in Table 7. Enablers to the use of both products included psychological capabilities, such as their perceived ability to offer protection from malaria to one’s family and community, as well as their perceived efficacy where participants could immediately observe the absence of mosquitoes near the VPSR product and a reduction in mosquito bites when wearing ITC. Ease of use was another major enabler to product uptake, mapped to physical capability and automatic motivation. The odour profile of the products was an important factor affecting user experience, where participants equated product odours with efficacy and safety. This presented a barrier to uptake affected by psychological capability and automatic motivation, where users need to understand that the VPSR is odourless yet effective, and that the smell of the ITC are safe. A barrier to uptake is also the ability of users to purchase the products, as they may not have the resources available to do so.

**Table 7:** Key informant comparison between the two products piloted

Theme	Quotes	COM-B theme
The efficacy of the VPSR product provides complete protection from mosquito bites, whereas I still got bitten by mosquitoes while wearing ITC, especially on uncovered parts of my skin	“*[The VPSR] is more efficacious than treated clothes.”*—Male forest ranger, age 24 *“When we have this [VPSR] product, mosquitoes are not coming or biting us*… *[When wearing treated clothes, on the uncovered treated clothing [on] my skin, mosquitoes still come to bite.*”—Male forest goer, age 26 “*There are more mosquitoes when not wearing the treated clothing, less mosquito bites when wearing it*.”—Male forest goer, age 23	Enabler for VPSR: psychological capability Users see that the product is efficacious Barrier for ITC: psychological capability and physical opportunity Target users do not understand that ITC still work even if you see mosquitoes nearby or landing on the clothing, and that additional protective methods will be needed for uncovered skin
The ITC has a smell and causes some side effects that I was not comfortable with	“*[I didn’t use the product because it smells bad and caused feel not well. Don't like it and do not want to wear it*.”—Female forest dweller, age 40 “*Yes, the treated clothes have the smell a little bit but just a minor thing. It does not affect anything to me, the main thing is to prevent mosquito bites and reduce malaria infection*.”—Male forest ranger, age 24 “*Strong smell and other people get rash when touching it. [People might be concerned about using this product because they] are afraid it will make them sick*.”—Male forest goer, age 31	Barrier for ITC: psychological capability and reflective motivation The treated clothes have an unpleasant smell and can cause side effects for some users. Target users do not understand that these effects are temporary and not serious
The ITC need to be treated periodically, hung up to dry, then worn. Watching the project staff treat my clothes, I think it looks easy and that I can do this myself	“*Not difficult, I can do it.*”—Male forest goer, age 26	Enabler for ITC: psychological capability Perception that retreatment of ITC is not difficult to do, but this needs to be tested
The VPSR product is best for staying in one place, especially because the product piloted was designed for indoor use and cannot get wet	“*Both are good, [the VPSR] is for one place, treated clothes is for moving around*.”—Male forest ranger, age 51 “*For the farm or the forest, use treated clothes because they can be used if wet. At home use [the VPSR], it lasts a long time compared to other insecticides.*”—Female forest dweller, age 55 “*I put [the VPSR product] in the plastic bag inside my backpack and keep it safe from the water*.”—Male forest ranger, age 37	Barrier for VPSR: physical capability and physical opportunity Product was not designed to use in wet conditions and target users do not have a way to keep the product dry for use in environmental conditions Enabler for VPSR: psychological capability Users perceive that the product has a longer duration of efficacy than some other malaria prevention products
The treated clothes are best for using when going to the forest, because it is easy to use effective when it rains	*“For the farm / chamka in the forest, treated clothes preferred because [the VPSR] cannot be used in the rain.”*—Female forest dweller, age 55 “*Clothes are easy to use and prevent being bitten, [the VPSR] you need to carry*.”—Male forest goer, age 38 *“Treated clothes [were the] best [because they] can be used when wet, it rained every day for forest patrol.”*—Male forest ranger, age 51	Enabler for treated clothes: physical capability and physical opportunity ITC were easy to use in the rainy outdoor conditions common for target users, and suitable for the mobile outdoor nature of their work

When comparing the two products piloted, participants perceived distinct advantages that drew insights on use case scenarios for each, with participants concluding that the VPSR product was best for staying in one place, particularly in indoor settings, while ITC were best for use in the forest due to ease of use for mobile work and continued efficacy in rainy conditions (Table 7). Drawing on differences between the products, the VPSR had more visible perceived efficacy from the lack of mosquitoes around the product as compared to ITC, which only prevents mosquito landing on treated clothes. Therefore, barriers to ITC include the need for psychological capability of users to understand that these clothes do not protect uncovered skin, as well as a need for physical opportunity to access additional protective measures for uncovered areas, such as topical repellents.

The ITC had a smell and reported side effects, which target users need the psychological capability to understand are temporary and not serious. Users also need to understand the need for periodic retreatment of clothes which participants believed would be easy to do based on watching study team members do so. Despite these differences, the ITC was cited to be easier to use in mobile forest settings due to ease of use and its ability to withstand rain. The VPSR would need to be carried when being mobile and could not get wet because it was designed for indoor use, presenting physical capability and opportunity as barriers for finding a way to keep the product dry in rainy conditions.

#### Recommended communications channels

Participants were asked how they would convince other people in their village to use either the VPSR product or ITC and mapped these onto BCW intervention functions (Table 8). Participants focused on accessing influencers, such as health centre staff, village health workers, and elders. Communications channels or touchpoints recommended for sharing the benefits of the products with others in their village were community meetings led by the village chief, and rangers also mentioned their monthly meetings being a useful place to introduce and promote new products. Billboards and motorcycle loudspeaker announcements were also suggested, while radio and mobile channels were not recommended given limited receptivity.

**Table 8:** Communications methods and channels recommended by key informants

Theme	Quotes	BCW intervention function
We have many influencers on malaria prevention practices, including using traditional methods, watching what other people in the community do, and advice from elderly community members and health center staff	“*[I learned] from the health center staff who educated me, I watch others and follow.”*—Male forest goer, age 23 “*I used to raise cattle; I used to burn the leaves or rice hay to get the smoke to prevent the mosquitoes from biting the cattle. I know this method from the old people*.”—Male forest ranger, age 55	Modeling: watching others use the product Persuasion: Listening to elders Education: Learning about additional malaria prevention practices
Community or ranger meetings are a recommended forum to introduce products particularly if influential leaders or experts, e.g., VMWs or ranger team leaders are involved	“*[I recommend you share information on these products at a] community meeting, invite villagers to join, let them ask questions and rest up their concerns. When they know the product well, they will use it*.”—Female forest dweller, age 40 “*The best way [to introduce products] is for VMW to organize meetings with villagers to introduce products. People will use it when they know it is effective and trust VMW.*”—Male forest goer, age 46 “*[Introduce the project at] monthly meetings because everyone joins is and reports to the chief ranger. This is a great time to share and get everyone used to it*.”—Male forest ranger, age 37	Persuasion/Education: leaders introduce products to community in open forums, citing their benefits to health
Targeted mass communication channels that are accessible to target communities can be useful to inform the village of new products	“*If there is an ad on the mobile motor(bike) or announcement would be good. Everyone in the village knows.*”—Male forest goer, age 38 “*Broadcasting on a radio or the big billboard is good because everyone can see it, not only rangers but everyone also knows it*.”—Male forest ranger, age 24 “*For radio, some will listen and some not because it is not possible in the forest*.”—Male forest ranger, age 51	Persuasion/Education: Provide information so that the entire community can be engaged

The COM-B components for areas identified for key informant perceptions of malaria (Table 5) and of the products piloted (Tables 6 and 7) and recommended communications channels mapped onto intervention functions using the BCW (Table 8) were used to inform the selection of behaviour change techniques to address these target behaviours as well as modes of delivery for introducing the products. For this, the pilot scale used in this study was considered, as well as the hypothetical delivery of those products through longer-term efforts, which would require sustained use (Table 9).

**Table 9:** COM-B components linked to intervention functions from barrier and enabler analysis to motivate behavior change

Target behavior	Capability		Opportunity		Motivation		Selected BCW intervention function (s)	Selected behavior change technique	Selected mode of delivery
	Phys	Psyc	Phys	Soc	Ref	Auto			
Trial use of VPSR and ITC		x	x		x		Modeling, education, and persuasion	Instruction on how to use product, information about health as well as non-health benefits from the perspective of users	Face-to-face sessions reinforced with illustrated printed or digital (i.e. on tablets) materials
Sustained use of VPSR and ITC		x		x		x	Modeling, enablement, and education	Instruction on how to use product, information about health benefits and emotional benefits	Multi-media campaign using channels accessible to the target audience i.e. outdoor billboards, illustrated print or graphic digital tools, mobile motorbike announcements and community events led by health center staff and/or village malaria workers and the village chief, and for rangers, the team lead
		x					Training	Demonstration on how to use products, including retreatment of clothes	
				x			Persuasion, modelling		

*Phys* physical, *psyc* psychological*, soc* social*, ref* reflective*, auto* automatic

The priority COM-B components to leverage enablers and address barriers to trial use of these products included psychological capability, physical opportunity, and reflective motivation, which we mapped to modelling, education, and persuasion. For purposes of piloting the products, the behaviour change techniques used were effective, comprising of instructions on how to use the product and information on its benefits delivered through face-to-face sessions. Village and ranger leaders were involved in the recruitment process, allowing for effective persuasion that study team members providing the products were trustworthy.

For sustained use of these products, similar to trial use priority COM-B components should include psychological capability, mapped to education to ensure that target populations understand what the products are for and how to use them. Different from trial use however, social opportunity and automatic motivation should be leveraged to encourage regular use, which could center around their ability to protect community members, including vulnerable groups such as elders and young children. For these COM-B components, BCW intervention functions for use can include modelling and persuasion through targeted advertisements and trusted members of society could encourage uptake, enablement through service provision would ensure accessibility, and training focused on the retreatment of ITC could be necessary. For these intervention functions, behaviour change techniques from the results above and modes of delivery drawing from Table 8 could include multi-media campaigns using outdoor billboards, printed or digital graphic tools, mobile motorbike announcements, and community events led by health centre staff, village malaria workers, village chiefs, and lead forest rangers.

## Discussion

Forest malaria challenges malaria elimination efforts in many locations, and is responsible for many remaining pockets of transmission in Southeast Asia [13]. This is the first study that examines risk factors, experiences and perceptions after use of a VPSR and ITC in Cambodia. The COM-B model and BCW were applied to analyse qualitative insights related to use of these products, to inform forest malaria elimination programming strategies in Cambodia. This study found that the forest goers, dwellers, and rangers who participated in this study all understood mosquito bites to be dangerous and had gaps in protection during waking hours, especially in forest settings, and furthermore found both products piloted to be efficacious for preventing mosquito bites. For use cases, participants preferred the VPSR product when travel was not required, while ITC was preferred when going to the forest due to its ease of use in not having to carry anything separately, and its efficacy in rainy conditions.

COM-B analysis identified that key enablers for use of both products included psychological capability and automatic motivation about their perceived efficacy and ease of use, requiring no skill or preparation. Barriers to use included psychological capability and reflective motivation that the odour of ITC was sometimes perceived as being toxic and that it was unable to protect uncovered skin from mosquito bites, while for the VPSR product physical capability and physical opportunity would be necessary to find ways to keep the product dry if being used in rainy forest settings. Mapping these COM-B components to BCW intervention functions, education can be used to explain that the smell of ITC is safe and that additional protective measures are necessary on exposed skin, that the VPSR needs to be kept dry, and that both products offer protection from malaria and dengue for target users and members of their community, saving time and money seeking care as well as risks posed to employment. Enablement will be needed to ensure products are accessible and affordable to target users (willingness to pay was between $1.44 and $2.71 monthly), and modelling and persuasion through respected members of the community, such as village leaders, ranger team leads, and targeted advertisements will likely be necessary for longer-term use of these products.

These results could be applied to the design of social behaviour change communication (SBCC) strategies to support the introduction and uptake of these products, recommending the complete and continuous use of multiple mosquito bite prevention products as the best way to avoid losing valuable time and money due to a possible serious illness. To enable this level of protection, the continued use of LLINs, long-lasting insecticide treated hammock nets, and other preventive measures should be promoted alongside efforts to motivate the use of VPSRs inside and outside the house, and ITC when leaving the house to go to the forest. If the target population chooses to treat short-sleeved shirts or short pants with insecticides, the use of topical repellents to protect uncovered parts of their body should be encouraged [26]. Results from this study also suggest possible delivery channels for SBCC to achieve these communication objectives, which include face-to-face sessions reinforced with illustrated print or digital materials in local language, as well as multi-media campaigns using accessible channels to target audiences e.g., outdoor billboards, mobile motorbike announcements, and community events. Across channels, campaign spokespeople or characters featured in SBCC content could include respected elderly community members and leaders and/or health worker staff.

With regard to generalizability, findings from this study are consistent with broader themes identified through other research on forest malaria, including poor access to healthcare as a result of mobility, and association with poverty [27, 28]. These results reinforce the assessment that efforts to eliminate forest malaria would benefit from the use of multiple vector control interventions, especially those that can be used to reduce outdoor mosquito biting [9], that could also be used in combination with chemoprophylaxis [13]. Findings on the lifestyle habits of forest goers, dwellers, and rangers in Mondulkiri province however, are specific to those within the specific district and province in Cambodia where the research was conducted, with similar occupation, gender, and ethnic groups present, as confirmed by similar findings in a 2018 study conducted amongst 4,200 forest workers in the same province [5]. Even in villages in Mondulkiri province, where reported mosquito peak biting times were similar, participants reported to be in different locations during those times [29], suggesting they should be a different target group than those in our study. Other risk profiles in Cambodia could include illegal loggers, who had very different forest-going habits compared to participants from this study who did not use mosquito coils due to the fear of being detected [6], as well as mobile populations that create temporary encampments in the forest, such as those described in Stung Treng province [30].

For specific products piloted in our study, many other studies on the use of VPSRs and ITC have been published. For VPSRs, one study investigated their use in Mondulkiri Province, Cambodia, in 2013 for indoor use amongst Bunong villagers living near the border with Vietnam, with similar user acceptability to findings from this study, where almost all participants and users of the VPSR product perceived it to be useful and would be willing to use it again [29]. That study found that socioeconomic status did not have a significant effect on willingness to pay for VPSRs, which is consistent with findings from this study where forest rangers, who despite having a higher income and socioeconomic status than forest goers and dwellers, had a lower willingness to pay for these products perhaps because vector control tools are provided to them for free through their jobs. VPSRs were also preferred to mosquito coils because they are long-lasting and do not require frequent user activation [9], and furthermore do not cause smoke which can irritate end users and may be a risk factor for lung cancer [31, 32].

For the use of ITC among forest-going populations, only one other publication is available, which explored the use of an odourless permethrin-based product in 2015 amongst 234 rubber tappers in the Mon State in Myanmar [33]. The study population was comprised of migrants staying in plantation lodges overnight, who used similar malaria preventive measures to those reported in our study, including insecticide-treated nets, mosquito coils, and long sleeves. Although the Myanmar study used an odourless product with a different active ingredient for which resistance is increasingly reported globally [2], user acceptability assessments had similar findings to those in this study, with participants citing that they liked the ease of use and comfort of using ITC. This Myanmar study also made suggestions on SBCC strategies that were consistent with findings from this study in Cambodia, suggesting that these strategies incorporate promotional and educational messages delivered through billboards, with products becoming available in health centres and shops.

Importantly, the products piloted are not yet available for widespread distribution for public health use. VPSRs may soon be available for this indication, with a recent cluster randomized trial in Indonesia showing that a VPSR provided 31.3% protective effect for incident malaria infections, offering even higher levels of protection in clusters with higher malaria endemicity [34]. A cluster randomized trial amongst 2,907 households in Iquitos, Peru using a VPSR showed a protective efficacy of 34.1% against *Aedes-*borne diseases [35]. Unitaid is sponsoring a large multi-country trial of VPSRs, and additional evidence on its efficacy in preventing malaria and dengue fever is forthcoming [36]. Insecticide-treated clothing has long been used by military populations, and there is evidence available on their efficacy [37]. A cluster randomized trial on the effect of permethrin-based clothing treatment with topical repellents on the incidence of malaria is underway [38]. Although most studies on ITC have used permethrin, etofenprox, the active compound used in this study, has better wash resistance than permethrin [38].

This study also supports findings in a wider research project, that topical repellents could be useful when wearing ITC due to the latter’s inability to protect uncovered areas of skin. Topical repellents are already in use by many rangers, suggesting its acceptability amongst this at-risk population in Mondulkiri province. They have also been widely distributed in a study conducted in Ratanakiri Province, Cambodia, which showed that regular use can be challenging [39, 40] and may need to be encouraged using SBCC-type approaches. Topical repellents have otherwise shown to be efficacious, reducing the odds of malaria infection in a large study amongst 116 villages in Myanmar [26], and reducing both malaria infection rates and mosquito density when distributed to a refugee camp in Northeastern Kenya [41].

The growing evidence base on VPSRs and ITC suggests that new products within these classes may be available for public health use in the coming years. In the meantime, two areas of research should be prioritized. First, to accelerate Cambodia’s malaria elimination goals and following evidence of entomological protective efficacy among local vectors in Mondulkiri province, products within these classes can be delivered to more at-risk individuals using SBCC strategies, collecting implementation outcomes on factors that enable or prevent sustained uptake including willingness to pay for these products alone and in combination with other products. These bite prevention products can be given with several other protective measures against malaria including chemoprophylaxis [42, 43], to inform the evidence base on eliminating forest malaria. Second, pending validation from larger studies, these findings that the odour of these products influences user acceptability should be applied to the development of new mosquito bite prevention products or optimization of existing products, using ingredients and formulations that have an acceptable or desirable smell to target populations.

This pilot study had several limitations. This study had a small sample size, did not trial the use of topical repellents together with other products examined, and distributed ITC involving a member of the study team treating clothes for participants, which could be done for large-scale implementation but would be much more expensive than teaching participants how to treat clothes. However, this sample size was likely sufficient given consistent findings by study participants and similarities in responses on user experience amongst a subset of only 10 of these participants. This study also suggests that forest goers and dwellers might be suitable for integrating into a single risk profile since their results were similar. This will be explored in the final phase of the parent study, which enrolled more than 2000 participants, which will allow for the examination of whether these groups can be further segmented by gender, socio-economic status, and more. For ITC distribution, future work can focus on identifying potential barriers to treating clothing effectively, and how to encourage retreatment at appropriate times periodically.

## Conclusion

Malaria elimination strategies for Cambodian forest-exposed populations could benefit from the use of VPSRs, ITC, and topical repellents to protect exposed skin not covered by treated clothes. These pilot study findings can guide the design of tailored SBCC strategies for a larger study in the parent project, incorporating education and training on the ability of these products to protect people from malaria, how to correctly use them, and what to expect from their use including their odours or lack thereof, enablement to ensure access, and persuasion from targeted advertisements and trusted members of society. This is the first stage of iterative assessment of roll-out strategies which should continue when conducting larger research studies and implementation projects. In parallel, larger-scale studies should examine whether research and development efforts should indeed prioritize the creation of products that are rainproof and easy to use in forest settings, with favourable odour profiles for target users.

### Supplementary Information

**Additional file 1.** Pre-intervention questionnaire.

**Additional file 2.** Post-product testing questionnaire.

**Additional file 3. **Key informant interview guide.

## Data Availability

All data generated or analysed during this study are included in this published article and are available upon request.

## Abbreviations

BCW: Behaviour change wheel
CNM: National Center for Parasitology, Entomology, and Malaria Control
COM-B: Capability, opportunity, motivation and behaviour change
EPA: Environmental Protection Agency
ITC: Insecticide-treated clothing
LLIHN: Long-lasting insecticidal hammock nets
LLIN: Long-lasting insecticidal net
ODK: Open Data Kit
RAI: Regional Artemisinin-resistance Initiative
US: United States
VMW: Village malaria worker
VPSR: Volatile pyrethroid spatial repellent
