# Supplementary material for: Applying the COM-B behaviour change model to a pilot study delivering volatile pyrethroid spatial repellents and insecticide-treated clothing to forest-exposed populations in Mondulkiri Province, Cambodia
Source: Malar J. 2023 Sep 1;22:251. doi: 10.1186/s12936-023-04685-1 (PMC10472618; doi:10.1186/s12936-023-04685-1)
Supplement: Supplementary file 1 — Additional file 1. Pre-intervention questionnaire. [file 12936_2023_4685_MOESM1_ESM.pdf]

Pre-intervention questionnaire

BITE Project Cambodia
Questionnaire 1 Phase 1

Participant ID:________________
Moderator:___________________________________Note taker:_______________________________________
Date:_______________________ Start time:___________ End time:____________

SECTION A. HOUSEHOLD ROSTER			
Data collector: Please explain you are going to ask some questions about the household where the participants live currently, when not working on this study, and this includes temporary visitors, for example any visitors that were in the household last night.			
Participant Group:	Ranger____ Forest Dweller____ Forest Goer____ Mosquito Collector_____		
Question	Response	Codes	Skip to
A.1. What is your age?	(years)		If <18 years old, STOP
A.2. What is your gender?	Male	1	
	Female	2	
A.3a. What ethnic group do you identify with?	Khmer	1	
	Cham	2	
	Phnong/Bunong	3	
	Vietnamese	4	
	Kroeng	5	
	Tumpoun	6	
	Charay	7	
	Prouv	8	
	Kachak	9	
	Kavet	10	
	Lao	11	
	Lun	12	
	Other	13	
A.3b. Describe other			
A.4. Tell us about your fluency of the Khmer language. Can you understand spoken Khmer?	Yes	1	
	No	2	
A.5. Can you speak Khmer fluently?	Yes	1	
	No	2	
A.6. Can you read in Khmer?	Yes	1	
	No	2	
A.7. Can you write in Khmer?	Yes	1	
	No	2	
	Yes	1	

A.8. Tell us about your fluency in the Bunong language. Can you understand spoken Bunong?	No	2	
A.9. Can you speak Bunong fluently?	Yes	1	
	No	2	
A.10. Can you read in Bunong?	Yes	1	
	No	2	
A.11. Can you write in Bunong?	Yes	1	
	No	2	
A.12. Are you the head of the household? (the person who provides the main income to support the family)	Yes	1	If YES, skip to #14
	No	2	
A.13. What is your position in the family relative to the head of household?	Father	1	
	Mother	2	
	Daughter	3	
	Son	4	
	Grandparent	5	
	Cousin	6	
	Husband	7	
	Wife	8	
	Other	9	
	None	99	
A.14. What is the highest level of education that you have completed?	None	1	
	Some primary	2	
	Some secondary	3	
	Completed secondary	4	
	More than secondary	5	
	Completed High School	6	
	Vocational college/University	7	
A.15. Do you spend time in the forest?	Yes	1	
	No	2	
A. 16a. How many days do you spend in the forest per week, on average, during the dry season?			

A.16b. How many days do you spend in the forest per week, on average, during the rainy season?			
A. 16c. Who goes to the forest with you when you go?	Spouses	1	
	Children	2	
	Parents	3	
	Neighbors	4	
	Co-workers	5	
	No one	6	
A.17. Now I will ask you where you spend time for each part of the day, when you are at home and not working on this study. (Select all that apply). In the early morning (5-9 am) (not later in the morning) where do you usually spend your time?	1. Mainly inside the house<br>2. Mainly outside the house<br>3. Away from the house<br>4. I don't know<br>5. No response		
A.18. In the later morning hours (9 am-noon) where do you usually spend your time?	1. Mainly inside the house<br>2. Mainly outside the house<br>3. Away from the house<br>4. I don't know<br>5. No response		
A.19. In the afternoon (noon - 6 pm), where do you usually spend your time?	1. Mainly inside the house<br>2. Mainly outside the house<br>3. Away from the house<br>4. I don't know<br>5. No response		
A.20. In the evening (6-9 pm), where do you usually spend your time?	1. Mainly inside the house<br>2. Mainly outside the house<br>3. Away from the house<br>4. I don't know<br>5. No response		
A.21. In the nighttime (after 9 pm), where do you usually spend your time?	1. Mainly inside the house<br>2. Mainly outside the house<br>3. Away from the house<br>4. I don't know<br>5. No response		

A.22. What time do you wake up in the morning?	Before 07:00 am	1	
	Between 07:00 am and 09:00 am	2	
	After 09:00 am	3	
A.23. What time do you go to sleep?			
A.24. Have you been bitten by mosquitoes inside your house recently?	Yes	1	
	No (skip to E.11)	2	

	Don’t know (skip to E.11.)	3	
A.24a. When was the last time you were bitten by a mosquito inside your house?	Today	1	
	Yesterday	2	
	Earlier this week	3	
	Last week	4	
	Earlier this month	5	
	Longer than one month	6	
	I don’t know	7	
A.25. How often do you get bitten by mosquitoes inside your house, in general?	Never	1	
	Several times a week	2	
	Daily	3	
A. 26. At what time of day do you normally get bitten by mosquitoes inside your home?	Early morning (5am-9am)	1	
	Morning (9am – noon)	2	
	Afternoon (noon – 3pm)	3	
	Late afternoon (3pm – 6pm)	4	
	Evening (6pm – 9pm)	5	
	At night (after 9pm)	6	
	Don’t know	7	
A.27. Have you been bitten by mosquitoes while you are outside around your house recently?	Yes	1	
	No	2	If NO, skip to #A28
	Don’t know	3	
A.27a. When was the last time you were bitten by a mosquito while you are outside around your house	Today	1	
	Yesterday	2	
	Earlier this week	3	
	Last week	4	
	Earlier this month	5	
	Longer than one month	6	
	I don’t know	7	
	Very often	1	

A.28. How often do you get bitten by mosquitoes while you are outside around your house?	Sometimes	2	
	Rarely	3	
	Never	4	
A. 29. At what time of day do you get bitten by mosquitoes while you are outside around your house?	Early morning (5am – 9am)	1	
	Morning (9am – noon)	2	
	Afternoon (noon – 3pm)	3	
	Late afternoon (3pm – 6pm)	4	
	Evening (6pm – 9pm)	5	
	At night (after 9pm)	6	
	Don't know	7	

Now I would like to ask you some questions about where you sleep normally, when you are not doing this study.

A.30. What does your primary (most used) sleeping structure look like?	Enclosed room with walls and ceiling/roofing	1
	Ceiling and 2-3 walls	2
	Only ceiling	3
	Completely open	4
	Notes:	
A.31a. What is the material of the roof made of?	Bamboo/grass/thatch	1
	Plastic sheet	2
	Tarpaulin	3
	Wood	4
	Tin	5
	Other	6
	Notes:	
A.31b. Describe other		
A.32. Where do you normally sleep during the rainy season?	Forest	1
	Wet rice fields	2
	Plantations	3

	Villages	4
A.33. Where do you normally sleep during the dry season?	Forest	1
	Wet rice fields	2
	Plantations	3
	Villages	4
A.34a. Which items do you carry between home and the worksite when you to go the places you mentioned above?	Global Fund Forest Pack	
	Bed nets	
	Hammock nets	
	Sleeping mats	
	Blankets	
	Food	
	Water	
	Medicine	
	Other	
A.34b. Describe "Other"		
A.35. If you sleep in a structure in the forest, have mosquitoes bothered you?	Yes	
	No	

Section B. BITING PREVENTION METHODS

Now I am going to ask you some more questions about mosquitoes in and around your home in your community.

B.1. Do you do anything to prevent mosquito bites inside your house?	Yes	1	
	No	2	If No, skip to B.3.
	Don't know	3	
B.1b. If no, why not?			
B.2a. What do you do to prevent biting inside your house during daytime?	Boil water	1	
	Sleep under mosquito net	2	
	Use insecticide spray	3	
	Use skin repellent	4	
	Burn coil	5	
	Burn incense or wood	6	

	Use hammock net	7	
	Use window or door screen	8	
	Use long sleeve clothing	9	
	Use GF forest pack	10	
	I don't know	11	
	Other	12	
B.2b. Describe "Other"			
B.2c. What do you do to prevent biting inside your house during nighttime?	Boil water	1	
	Sleep under mosquito net	2	
	Use insecticide spray	3	
	Use skin repellent	4	
	Burn coil	5	
	Burn incense or wood	6	
	Use hammock net	7	
	Use window or door screen	8	
	Use long sleeve clothing	9	
	Use GF forest pack	10	
	I don't know	11	
	Other	12	
B.2d. Describe "Other"			
B.3. Do you do anything to prevent mosquito bites outside the house?	Yes	1	
	No	2	If No, skip to B.5.
	I don't know	3	
B.4a. What do you do to prevent mosquito biting outside the house or/and in the village during daytime?	Boil water	1	
	Sleep under mosquito net	2	
	Use insecticide spray	3	
	Use skin repellent	4	
	Burn coil	5	

	Burn incense or wood	6	
	Use hammock net	7	
	Use window or door screen	8	
	Use long sleeve clothing	9	
	Use GF forest pack	10	
	I don't know	11	
	Other	12	
B.4b. Describe "Other"			
B.4c. What do you do to prevent mosquito biting outside the house or/and in the village during nighttime?	Boil water	1	
	Sleep under mosquito net	2	
	Use insecticide spray	3	
	Use skin repellent	4	
	Burn coil	5	
	Burn incense or wood	6	
	Use hammock net	7	
	Use window or door screen	8	
	Use long sleeve clothing	9	
	Use GF forest pack	10	
	I don't know	11	
	Other	12	
B.4d. Describe "Other"			
B.5a. If you go to the forest/work site/rice fields/chamkar, what do you do anything to prevent mosquito bites during daytime?	Boil water	1	
	Sleep under mosquito net	2	
	Use insecticide spray	3	
	Use skin repellent	4	
	Burn coil	5	
	Burn incense or wood	6	
	Use hammock net	7	
	Use window or door screen	8	
	Use long sleeve clothing	9	

	Use GF forest pack	10	
	I don’t know	11	
	Other	12	
B.5b. Describe "Other"			
B.5c. If you go to the forest/work site/rice fields/chamkar, what do you do anything to prevent mosquito bites during nighttime?	Boil water	1	
	Sleep under mosquito net	2	
	Use insecticide spray	3	
	Use skin repellent	4	
	Burn coil	5	
	Burn incense or wood	6	
	Use hammock net	7	
	Use window or door screen	8	
	Use long sleeve clothing	9	
	Use GF forest pack	10	
	I don’t know	11	
	Other	12	
B.5d. Describe "Other"			
B.6. Would you like to use any other methods or products to prevent mosquito bites?	Yes	1	If No, skip to C.1.
	No	2	
	Don’t know	3	
B.7. What methods or products would you like to use to prevent mosquito bites, that you are not already using?	Specify:		
SECTION C: BED-NETS AND HAMMOCK NETS RECORD			

I would like to ask you about bed-nets that you have in your current household.

This includes all nets that you own and were in the household when you most recently stayed there.

First ask all questions for net 1, then for net 2, etc...

If there are more than three bed-nets in the household, use additional sheets.

Bed net questions:

C1. Do you have bed net?	Yes	1	
	No	2	If NO; Skip to C5
C.1a. How many bed nets does your household have?			
	Net 1	Net 2	Net 3
C.2a. (Bed net #1) Where did your household obtain the net?	1. Family/friend	1. Family/friend	1. Family/friend
	2. Government	2. Government	2. Government
	3. NGO	3. NGO	3. NGO
	4. Shop/Market	4. Shop/Market	4. Shop/Market
	5. Itinerant seller	5. Itinerant seller	5. Itinerant seller
	6. Other:	6. Other:	6. Other:
	7. Don't know	7. Don't know	7. Don't know
C.2b. How far away (in kilometers) was the shop/ market?			
C.2c. Describe “Other”			
C.2d. (Bed net #1) Did you have to pay for the net?	1. Yes	1. Yes	1. Yes
	2. No	2. No	2. No
	3. Don’t know	3. Don’t know	3. Don’t know
C.2e. How much did you pay (in Riel)?			
C.2f. (Bed net #1) Did anyone sleep under the bed net the last time you stayed in the house?	1. Yes	1. Yes	1. Yes
	2. No	2. No	2. No
	3. Don’t know	3. Don’t know	3. Don’t know
C.2f1.When was the last time the net was used?			

C.2g1. (Bed net #1) How many adults slept under the bed net that night?	-Adults________	-Adults________	-Adults________
C.2g2. (Bed net #1) How many children slept under the bed net that night?	-Children______	-Children______	-Children______
C.2h1. How many females slept under the net that night?			
C.2h2. How many males slept under the net that night?			
C.2i. (Bed net #1) Where was the net hung that night?			
**Hammock net** questions:			
C.5a. Do you have hammock net?<br>1. Yes<br>2. No (If NO; skip to D1a)			
C.5b. How many hammock nets does your household have?			
	Hammock net #1	Hammock net #2	Hammock net #3
C.6a. (Hammock net #1) Where did your household obtain the net?	1. Family/friend	1. Family/friend	1. Family/friend
	2. Government	2. Government	2. Government
	3. NGO	3. NGO	3. NGO
	4. Shop/Market	4. Shop/Market	4. Shop/Market
	5. Itinerant seller	5. Itinerant seller	5. Itinerant seller
	6. Other:	6. Other:	6. Other:
	7. Don't know	7. Don't know	7. Don't know
C.6a1. (Hammock net #1) Does your hammock net used was treated or not?<br><br>(If you check the label – CNM logo/GF hammock nets)	1. Yes	1. Yes	1. Yes
	2. No	2. No	2. No
C. 6b. (Hammock net #1) How far away was the shop/ market?			
C.6c. (Hammock net #1) Describe "Other"			
C.6d. (Hammock net #1) Did you have to pay for the net?	1. Yes	1. Yes	1. Yes
	2. No	2. No	2. No

	3. I don't know	3. I don't know	3. I don't know
C.6e. (Hammock net #1) How much did you pay (in Riel)?			
C.6f. (Hammock net #1) Did anyone sleep under the hammock net last time you stayed in the house?	1. Yes	1. Yes	1. Yes
	2. No	2. No	2. No
	3. Don't know	3. Don't know	3. Don't know
C.6f1.When was the last time the hammock net was used?			
C.6g. (Hammock net #1) What was the age of the person who slept under the net that night?			
C.6h. (Hammock net #1) What was the gender of the person who slept under that night?	1. Female	1. Female	1. Female
	2. Male	2. Male	2. Male
	3. I don't know	3. I don't know	3. I don't know
C.6i. Where was the net hung that night?			

Section D: Clothing		
I would like to ask you some questions about the clothes you wear and how you wash them.		
D.1a. How many Long Pants do you own and wear?		
D.1b. How many Short Pants do you own and wear?		
D.1c. How many Long Sleeves Shirts do you own and wear?		
D.1d. How many Short Sleeve Shirts (including T-Shirts) do you own and wear?		
D.1e. How many Dresses/ Skirts do you own and wear?		
D.1f. How many Krama do you own and wear?		
D.1.g. How often do you need to replace your shirts, both long and short (whether because they are old, or torn, or soiled, or for another reason)?		

D.1.h. How often do you need to replace your pants, both long and short (whether because they are old, or torn, or soiled, or for another reason)?		
D.2. Which do you wear into the forest during the day?	Long Pants	1
	Short Pants	2
	Long Sleeve Shirts	3
	Short-Sleeve Shirts (including T-shirts)	4
	Dresses/skirt	5
	Krama	6
	Do not wear top clothing or shirt	7
D.3. Which do you wear into the forest during the night?	Long Pants	1
	Short Pants	2
	Long Sleeve Shirts	3
	Short Sleeve Shirts (including T-shirts)	4
	Dresses/Skirt	5
	Krama	6
	Do not wear top clothing or shirt	7
D.4a. How often do you wash your clothes during the dry season?	Every day	1
	Every 2-3 days	2
	At least once a week	3
	Less than once a week	4
	Never Wash	5
D.4b. How often do you wash your clothes during the wet season?	Every day	1
	Every 2-3 days	2
	At least once a week	3
	Less than once a week	4
	Never Wash	5
	Notes:	
D.4c. Notes on washing frequency:		
D.5a. What do you use to wash your clothes?	Soap	1

	Detergent powder	2
	Bleach	3
	None, water only	4
	Notes:	
D.5b. Notes on what is used to wash clothes:		
D.6a. Where do you wash your clothes?<br><br>(multiple answer)	River/Stream	1
	Bucket with well water	2
	Bucket with rain water	3
	Other	4
	Notes:	
D.6b. Describe "Other"		
D.6c. Notes on where clothes are washed		
D.7a. If you use a container/ bucket to wash clothes, where do you put the used water?	Pit latrine	1
	Ground	2
	Stream or river	3
	Well or bore-hole	4
	Other:	5
D.7b. Notes on where used water is put:		
D.8a. How do you scrub the clothing when washing then?	By hand only	1
	Against hard surfaces (e.g. rocks, wood)	2
	Other	3
	Notes:	
D.8b. Describe "Other":		
D.9. How do you dry clothing after washing at home?	Under shade	1
	In open sunlight	2
D.10. How do you dry clothing after washing at worksite?	Under shade	3
	In open sunlight	4

Section E	Question	Outcome	Code

E	1a	Which of the following activities has been a source of income for you/your family in the past year?	Farmer	1
			Market trader	2
			Forest collector/forager	3
			Ranger	4
			Driver/ motorbike taxi	5
			Day laborer	6
			Indigenous leader	7
			Handicraft (basket weaving etc.)	8
			Retired	9
			Unemployed	10
			Other	11
E	1b	Describe "Other":		
E	2	Do you have phone network coverage at your house/station?	Yes	1
			No	2
			Don't know	3
E	3	Do you have internet coverage at your house/station?	Yes	1
			No	2
			Don't know	3
E	4	What are the walls made from in your home/station?	Mud/ mud with reinforcing bamboo or straw	1
			Wood/ tree bark	2
			Palm/ bamboo/ thatch	3
			Brick	4
			Cement	5
			Plastic	6
			Metal Sheeting	7
			Don't know	8
E	5a	How many rooms are in your primary living structure?	1 room	1 Skip to E6
			2 rooms	2
			3 rooms	3
			4 rooms	4
			5 rooms	5
			≥6 rooms	6
E	5b	How many of these rooms are used for sleeping?		
E	5c	What type of material separates the rooms in your primary living structure?	Plastic Sheet	1
			Bead Strings	2
			Bamboo	3
			Thatch	4
			Mud	5
			Wood	6
			Brick	7
			Metal	8
			Other	9
E	5d	Describe "Other"		
E	6	How close is your main (primary) house house/ranger station to the forest?	I live in the forest	1
			Within 100m	2
			Within 200m	3
			Within 500m	4
			Within 1 km	5
			Within 1-2 km	6
			Over 2 km	7
E	7	Do you purchase items for your household outside of your village?	Yes	
			No	

			Don't know	
E	8a	What type of items do you normally purchase at these places outside of the village?	Electronics (TV, radio)	1
			Food	2
			Kitchen Items	3
			Farm related supplies	4
			Health related supplies	5
			Mobile phone and accessories	6
			Mosquito protection supplies (coils, nets, repellents, etc.)	7
			Other	8
			Specify	
E	8b	Describe "Other"		
E	9	How much money (Riel) do you spend on mosquito prevention supplies per month?		
E	10	How far do you travel to buy things (km from household)?		
E	12	Do you watch television (even at someone else's house, at a place of business, or elsewhere)?	Yes	1
			No (Skip to E15)	2
E	13a	Where do you watch television?	In my home	1
			At village Chief's house	2
			At friend's house	3
			At a business	4
			Other	5
E	13b	Describe "Other"		
E	14	How often do you watch television?	Once per week	1
			2-3 times per week	2
			4-5 times per week	3
			Every day	4
E	15	Do you watch videos or movies on the Internet, such as on YouTube?	Yes	1
			No (Skip to E18)	2
E	16a	Where do you watch Internet videos or movies?	In my home	1
			At village Chief's house	2
			At friend's house	3
			At a business	4
			Other	5
E	16b	Describe "Other":		
E	17a	On what device do you watch internet videos or movies?	Computer	1
			Tablet	2
			Mobile phone	3
			Other	4
E	17b	Describe "Other":		
E	18	Do you listen to the radio?	Yes	1
			No (Skip to F1)	2
E	19a	How do you listen to the radio?	Radio set	1
			Mobile phone	2
			Tablet	3
			Computer	4
			Other	5
E	19b	Describe "Other":		
E	20	What time of day do you listen to the radio?	Early morning	1
			Morning	2
			Noon	3
			Afternoon	4

			Evening	5
			Night	6
E	21	What channels do you listen to on the radio?	Please specify (free text)	
Section F	Question		Outcome	Code
F	1	[Please tell me whether you agree or disagree with following statement:] Mosquitoes are dangerous	Agree	1
			Disagree	2
			Neither agree or disagree	3
F	2	What can happen if you get bitten by mosquitoes? … Serious diseases (severe, needing hospitalization)	Agree	1
			Disagree	2
			Neither agree or disagree	3
F	3	What can happen if you get bitten by mosquitoes? … Non-serious health issues (feeling ill for a few days)	Agree	1
			Disagree	2
			Neither agree or disagree	3
F	4	What can happen if you get bitten by mosquitoes? … Itching	Agree	1
			Disagree	2
			Neither agree or disagree	3
F	5	What can happen if you get bitten by mosquitoes? … Ugly marks on skin	Agree	1
			Disagree	2
			Neither agree or disagree	3
F	6a	What worries/concerns you about mosquito bites?	Notes:	
F	6b	Why does this worry you?		
F	7a	How many times have you been diagnosed with malaria?	Notes:	
F	7b	How many people in our household have ever been diagnosed with malaria?	Notes:	
F	7c	How many times have OTHER PEOPLE in your household been diagnosed with malaria, in total?		
F	8	When was the last time someone in your household was diagnosed with malaria?	Last Week,<br>Last Month,<br>Last 3 Months,<br>Last 6 Months,<br>Last Year,<br>More than one year	1<br>2<br>3<br>4<br>5<br>6
F	9	How and where were you or the person in your household diagnosed with malaria?		
F	10	The last time you or your family member had malaria, what was done to recover?		
F	11a	The last time you or someone in your household was sick [with any illness], where did you go?	Health Centre	1
			Referral hospital	2
			Private clinic	3
			Pharmacy	4
			Traditional healer	5
			MMW or VMW	6
			Other	7
F	11b	Describe “Other”:		
