# Supplementary material for: Applying the COM-B behaviour change model to a pilot study delivering volatile pyrethroid spatial repellents and insecticide-treated clothing to forest-exposed populations in Mondulkiri Province, Cambodia
Source: Malar J. 2023 Sep 1;22:251. doi: 10.1186/s12936-023-04685-1 (PMC10472618; doi:10.1186/s12936-023-04685-1)
Supplement: Supplementary file 2 — Additional file 2. Post-product testing questionnaire. [file 12936_2023_4685_MOESM2_ESM.pdf]

# Post-product testing questionnaire

G1 Intervention Product
G2a Do you know what the specific use of this product is?
G2b Describe "Other"
G3a How long do you think this product lasts, or is effective? Why do you think that?
G4a Did you notice any changes in the number of mosquito bites after you used the product, compared to normal?
G4b Describe "Other"
G5a Have you had any side effects or discomfort with the product?
G5b Describe "Other"
G6a If you were to compare this product to bed-nets, would you prefer using this product for mosquito-bite prevention?
G6b If yes, why?
G6c If no, why?
G7a Would you prefer using this product over hammock nets for mosquito-bites prevention?
G7b If yes, why?
G7c If no, why?
G8a Would you prefer using this product over skin repellents or mosquito coils for mosquito-bites prevention?
G8b If yes, why?
G8c If no, why?
G9 How would you rate this product overall?
G10a Would you continue to use this product, if you had access?
G10b If no, why?
G11 Would you recommend this product to others?
G12a Would you be willing to pay for this product on a monthly basis?
G12b If yes, how much would you be willing to pay per month (in Riel)?
G12c Where would you prefer to be able to purchase this product?
G12d If in your nearest market, how far away is the market (km)?
G12d Describe "Other"
