# Supplementary material for: Applying the COM-B behaviour change model to a pilot study delivering volatile pyrethroid spatial repellents and insecticide-treated clothing to forest-exposed populations in Mondulkiri Province, Cambodia
Source: Malar J. 2023 Sep 1;22:251. doi: 10.1186/s12936-023-04685-1 (PMC10472618; doi:10.1186/s12936-023-04685-1)
Supplement: Supplementary file 3 — Additional file 3. Key informant interview guide. [file 12936_2023_4685_MOESM3_ESM.pdf]

Key Informant Interview Guide.
Topic Guide for Forest Rangers, Forest Dwellers, and Forest Goers
Mondulkiri, Cambodia
Facilitator: …………………………………. Note taker: …………………….. Date: ………………………………..
Time……………………………………… Village………………………. Participant ID……………………………..
GPS Location………………………………
Target Group (circle one): 1) Forest Ranger 2) Forest Dweller 3) Forest Goer
Gender:
Age:

INTRODUCTION

Hello, my name is *[INTERVIEWER'S name] and this is* [*NOTE-TAKER'S name].* We want to thank you for agreeing to take part in this interview. I will be leading the interview and *[NOTE-TAKER'S name]* will be taking notes.

Before we get started with the interview, I would like to show you this informed consent form which you have previously signed.

Do you have any questions about the informed consent?
May I have your permission to turn on the audio recorder and begin the interview?

[TURN ON AUDIO RECORDER if permission granted]

[NOTE-TAKER start recording]

MALARIA RISKS AND PREVENTION TOOLS

Do you know about malaria?
Have you ever had malaria before?
How many times have you ever had malaria before?
Do you feel that you are at risk of malaria infection?
Why or why not?
How big of a risk do you think malaria is to you? Why?
Do you worry about getting malaria?
Do you feel that your community is at risk of malaria infection?
Why or why not?
How big of a risk do you think malaria is to your community? Why?
Do you worry about people in your community getting malaria?
Do you feel that many people in your community get malaria?
Why or why not?
Have you noticed any changes with malaria over the past few years? For example, maybe there have been more or fewer infections? What kind of changes have you seen? What kind of changes? Probe for changes in number of people with fever and malaria in the last 3 or 5 years, malaria knowledge and control, personal protection practices, bednet use.
Do you know the signs and symptoms of malaria? What are the specific signs/symptoms of malaria that tell you when someone has a malaria infection?
Which group of people in the community do you think gets malaria most often?
Why do you think these people get malaria more than others?
Do you know about methods for protecting yourself from mosquito bites? What are they?
How did you learn to prevent mosquito bites?
What do people normally do to protect themselves or their families from getting malaria?
What do people like about these methods? And what do they dislike about them?

MALARIA PRODUCTS – GENERAL EXPERIENCE

Over the last three weeks, you were asked to try three different malaria prevention products [List the products and show bottles.]
[Confirm with participant that they were given the products.]

MALARIA PRODUCT EXPERIENCE: #1 PIRK

Did you receive PIRK?
Did you use this product during the last week?
If no, SKIP to question 23. (to be changed to 22, there was a repeat question)
How many days do you remember using it? (record as number of days used out of number of days they had it)
Can you tell me how/when/where you used it?
For example, in the farm, village, or forest?
During the day or at night?
Where did you place it?
Did you bring it with you when you were going to the forest?
If yes, how did you carry/bring it with you to the forest?
Did you do anything to remind yourself to use it?
Do you feel that it was easy or hard to use? Why? [Probe: Any challenge with applying it? Any challenge with children around it? Any challenge with maintenance of it?]
Do you feel it was helpful to reduce mosquitoes?
Why or why not?
Probe: When you are outside? Or when you went to the forest?
Probe: The time (night/day) & locations - inside or outside home; in forest, farm huts or other places?
What did you think about the way it smelled? Probe: like/dislike, if disliked how could the smell be better?
What did you think about the way it looked (packaging/shape)? Probe: like/dislike, how could package be better?
Do you have any concerns about safety when using this product?
If yes, what are your concerns? And why?
Do you think other people in this community would have any concerns about safety when using this product?
What might those concerns be?
What did you like most about this product (e.g. compared to others)?
Is there anything you didn't like about it?
What would you tell your neighbor/forest going mate to convince him to try the product for the first time, based on your experience? Probe: what is the main benefit of using this product in your opinion?
For those who did not use the product at all:
If you didn't use this product, can you tell me why you didn't use it?
For all:
Do you have any questions or comments for us about this product? I may not be able to answer everything, but I will try! (record questions)

MALARIA PRODUCT EXPERIENCE #3: Etofenprox

Did you use this product during the last week?
If no, SKIP to question 23.
How many pairs of your clothes would you like to be treated with Etofenprox insecticide?
How many days do you remember wearing the treated clothing? (record as number of days used out of number of days they had it)
Can you tell me how/when/where you used the treated clothing?
For example, in the farm, village, or forest?
During the day or at night?
Where did you wear the treated clothing?
Did you bring treated clothing with you when you were going to the forest? (or did you wear the treated clothing with you when you were in the forest)
If yes, how did you carry/bring it with you to the forest? Or did you wear it?
Did you do anything to remind yourself to wear the treated clothing?
Do you feel that it was easy or hard to use? Why? [Probe: Any challenge with children around it? Any challenge with maintenance of it?]

Probe: were there any allergies with children touching or wearing the treated clothing? Any concerns of children wearing treated clothing? If yes, why?
Any concerns about the limited physical coverage of treated clothing, for example being short sleeved and shorts/skirts with exposed skin on legs and arms. Were you bitten by mosquitoes on the exposed legs, arms or body parts not covered by the clothing?"

You saw the project staff treat your clothing. Do you think it looked difficult to do?
Do you feel the treated clothing was helpful to reduce mosquitoes?
Why or why not?
Did you notice any difference on the days or nights that you did not wear treated clothing? Can you describe them?
What did you think about the way the treated clothing smelled? Probe: like/dislike, if disliked how could the smell be better?
What did you think about the way the treated clothing looked? Did the clothing look any different after it was treated?
Do you have any concerns about safety when using this product?
If yes, what are your concerns? And why?
Do you think other people in this community would have any concerns about safety when using this product?
What might those concerns be?
What did you like most about this product (e.g. compared to others)?
Is there anything you didn't like about it?
What would you tell your neighbor/forest going mate to convince him to try the product for the first time, based on your experience? Probe: what is the main benefit of using this product in your opinion?
For those who did not use the product at all:
If you didn't use this product, can you tell me why you didn't use it?
For all:
Do you have any questions or comments for us about this product? I may not be able to answer everything, but I will try! (record questions)

PRODUCT COMPARISON

Now I am going to ask some questions about both products. I'd like to know what you think when you compare the two products.

If you had to choose only one of these products to use, which one would you choose? Why? (prompt: what did you like best about the product you would choose?)
Which product would you be the most likely to use consistently when you go to the forest? Why?
Which product do you think is easiest for people like you to use? Why?
If you compare the two products, which one do you think is most effective to prevent mosquito bites? Why?
Thinking again about the two products, which one do you think people who spend time in the forest would like to use more? Why?
Thinking back to your preferred product (answer to Q1), if we asked you to convince another person from your village to use this product when s/he visits the forest, what would you tell him/her about the product to convince them to use it?
If we could share information about the benefits of this product to other people in your village, which channels do you think would be the most effective?
(wait for answer and then probe: radio? outdoor signs? meetings led by village chief? an in-house reminder (i.e. sticker on a mirror close to the family hygiene station)? Any other ideas?
What do you think would help motivate people to use these products consistently when they are at risk of mosquito bites?
Anything else you'd like to mention?

Thank you so much for your information and participation.
